# IUC24432-80 Neoadjuvant or induction chemotherapy (N/IC) for bladder cancer: updated “real-world” experience from Croatia

**DOI:** 10.1093/oncolo/oyaf248.028

**Published:** 2025-08-29

**Authors:** J Murgic, M Jazvic, I Tomaskovic, P Bokarica, M Gamulin, I Dilber, M Sambic Penc, D Zahirovic, T Omrcen, A Frobe

**Affiliations:** Department of Oncology and Nuclear Medicine, Sestre milosrdnice University Hospital Center, Zagreb, Croatia; Department of Oncology and Nuclear Medicine, Sestre milosrdnice University Hospital Center, Zagreb, Croatia; Department of Urology, Sestre milosrdnice University Hospital Center, Zagreb, Croatia; Department of Urology, Sveti Duh University Hospital, Zagreb, Croatia; Department of Oncology, Zagreb University Hospital Center, Zagreb, Croatia; Department of Oncology, General Hospital Zadar, Croatia; Department of Oncology, Osijek University Hospital Center, Osijek, Croatia; Department for Tumors, Rijeka University Hospital Center, Croatia; Department of Oncology, Split University Hospital Center, Split, Croatia; Department of Oncology and Nuclear Medicine, Sestre milosrdnice University Hospital Center, Zagreb, Croatia

## Abstract

**Background:**

Despite level I evidence and known overall survival (OS) benefit of cisplatin-based neoadjuvant chemotherapy (NAC) for muscle-invasive bladder cancer (MIBC), its use remains suboptimal and varies geographically. We report treatment patterns and outcomes in patients (pts) receiving N/IC within the Croatian Uro-Oncology Network.

**Methods:**

We retrospectively reviewed records of consecutive patients treated with N/IC. Endpoints included pathologic complete response (pCR, ypT0N0), major pathologic response (MPR, ypT ≤ 1N0), disease-free survival (DFS), and OS. Survival was estimated from the N/IC start using Kaplan–Meier and log-rank tests. Clinical variables were assessed via Cox regression.

**Results:**

A total of 289 patients received N/IC across 9 sites. Clinical stage: II (45%), IIIA (29%), IIIB (24%). Median age was 66 years (range 40-83); 76% were male; 18% had ECOG PS 1; 23% had histologic variants. Regimens included cisplatin/gemcitabine (70%), dose-dense MVAC (24%), carboplatin/gemcitabine (3%), and cisplatin/etoposide (2%). Median duration was 2 months; median 4 cycles. Grade ≥3 toxicity occurred in 45%, including neutropenia (G3 18%, G4 10%), anemia (G3 7%), thrombocytopenia (G3 13%), and mucositis (G3 7%).

Cystectomy was performed in 226 patients (78%). The median time from N/IC start to surgery was 4 months; from N/IC end to surgery, 2 months. Reasons for non-surgical management included progression (*n* = 13), refusal (*n* = 14), bladder preservation (*n* = 8), or ECOG deterioration (*n* = 4). Among operated patients, pCR and MPR were achieved in 23% and 38%, respectively. After a median follow-up of 35 months, 34% had a recurrence (22% distant, 7% locoregional, 6% both). Median DFS was 33 months (95% CI, 15-56), and OS was 41 months (95% CI, 25-91). On multivariable analysis, only pCR was independently associated with improved DFS (HR 0.2; *P* < .0001) and OS (HR 0.3; *P* = .0002). Nine pts (4%) received adjuvant radiotherapy; none received adjuvant nivolumab.

**Conclusions:**

In this national real-world cohort, pCR was associated with improved survival, consistent with clinical trial data. Broader adoption of N/IC will require multidisciplinary care and structured patient counseling.

